# The relationship between the secondary implant stability quotient and oxidized implant-related factors: A retrospective study

**DOI:** 10.1016/j.heliyon.2024.e39156

**Published:** 2024-10-10

**Authors:** Fawaz Alzoubi, Abdulkareem Abdullah Alhumaidan, Hamad Saleh AlRumaih, Firas Khalid Alqarawi, Omar Omar

**Affiliations:** a Department of General Dental Practice, Faculty of Dentistry, Kuwait University, Kuwait; b Department of Preventive Dental Sciences, College of Dentistry, Imam Abdulrahman bin Faisal University, Dammam, Saudi Arabia; c Department of Substitutive Dental Sciences, College of Dentistry, Imam Abdulrahman bin Faisal University, Dammam, Saudi Arabia; d Department of Biomedical Dental Sciences, College of Dentistry, Imam Abdulrahman bin Faisal University, Dammam, Saudi Arabia

**Keywords:** Resonance frequency analysis, Dental implants, Osseointegration, Implant design, Regression analysis

## Abstract

**Objective:**

The present retrospective study aimed to determine the relationship between the secondary implant stability quotient and different parameters related to an oxidized implant.

**Methods:**

A total of 135 patients (305 oxidized implants) were included. Implant-related factors (length, diameter, surgical and loading protocols, grafting, insertion torque, and primary stability) were used for comparisons and linear regression analyses, using secondary ISQ as the dependent variable.

**Results:**

At the patient level, the mean time from implantation to secondary ISQ registration was 20.3 ± 29 weeks, and the mean secondary ISQ was 77.30 ± 7.22. The ISQ did not reveal significant differences regarding implant lengths, loading protocol, and simultaneous grafting. In contrast, platform diameters (3.5, 4.3, and 5.0), surgical protocols (one stage versus two stages), insertion torque (<35 Ncm versus >35 Ncm), and primary stability (achieved versus not achieved) all revealed significant secondary ISQ differences. Nevertheless, the regression analysis demonstrated that the platform diameter was the only variable significantly and positively predicted the secondary ISQ. Similar findings were found with the implant level analysis.

**Conclusions:**

Among different implant- and protocol-related parameters, the platform diameter of the oxidized implant appears to be the only significant predictor of high secondary ISQ values at the time of superstructure connection.

## Highlights

• Bivariate comparisons reveal several differences in the secondary ISQ values.
• Narrow implant, one-stage protocol, and low insertion torque are associated with low secondary ISQ.
• According to regression analysis, only platform diameter provides a significant prediction for ISQ.
• The study adds to the debate on what RFA implies concerning osseointegrated implants.

## 1 Introduction

Osseointegrated dental implants represent a well-documented and highly successful treatment for edentulous patients [[1], [2], [3]]. The definition of osseointegration has evolved from “A direct contact between living bone and implant at the light microscopic level” [4] – reflecting the histological picture – to “A structural and functional connection between ordered living bone and the surface of load-carrying implant” [5] – incorporating the loading condition into the definition. Eventually, the definition was revised to “The stable anchorage of an implant achieved by direct bone-to-implant contact” [6], which attributes the implant stability to the histological picture of a direct interface between an implant and bone.

Clinically, these definitions translate into an absence of mobility or, in other words, a functionally stable implant in the recipient bone [7,8]. Implant stability is further classified into primary and secondary, based on the time from implant installation to the establishment and maintenance of osseointegration [9,10]. Primary stability is recorded at the time of installation, and it is considered merely a physical phenomenon comprising the frictional contact between the implant and the recipient bone [11]. Over time, the osseointegration process evolves, governed by a series of biological events consisting of an initial inflammatory response and consecutive phases of bone formation and remodeling [12,13]. These events account for the gradual reduction in primary stability and the subsequent replacement with the secondary biological stability dependent on the deposition and maintenance of a mature bone onto the surface of the titanium implant [12].

Over the history of osseointegration, several methods have been used to evaluate implant stability, from the clinical perception of resistance during implant insertion, through judgments based on percussion tests, reverse and insertion torques, and eventually via the application of the damping capacity assessment (periotest) and the resonance frequency analysis [9,14]. Among all these, RFA has become the most acceptable and widely used technique for the clinical measurement of oral implant stability, accredited mainly to its objectivity, simplicity, and noninvasiveness [15,16]. In principle, RFA-based devices use a piezoelectric transducer emitting magnetic pulses to a magnetic peg that is screwed into the implant to make it vibrate [14,17]. The implant resistance to such vibration is converted and expressed as an Implant Stability Quotient (ISQ) on a scale of 1–100, with the higher the values reflecting the higher the stability.

Resonance frequency analysis applies a bending force and measures the stiffness of the implant-bone junction rather than the level of implant-bone contact [18,19]. Hence, RFA is affected mainly by the bone density surrounding the implant, where the denser the recipient bone is, the higher ISQ values of primary stability [19]. Extensive research explored the effects of implant- and procedure-related factors on the secondary ISQ measurements (secondary stability). These include the implant location and bone quality [[20], [21], [22], [23]], surgical procedures [[24], [25], [26]], insertion torque [[27], [28], [29]], loading protocol [20,30,31], need for grafting [32], and implant surface, design and dimensions [20,[33], [34], [35], [36], [37], [38]]. However, variable and sometimes contradictory results have been reported, suggesting that only some factors may influence the secondary ISQ results. Moreover, a limitation is that most studies have mainly used comparative or simple univariate correlational approaches [39,40], which do not consider the confounding effects of the different parameters on the secondary stability. Using implants with variable designs and surfaces represents another consideration, which limits cross-comparisons and generalization of the findings for each specific implant type.

Nowadays, oral implant surfaces are modified to promote faster and stronger fixation in the recipient bone. Anodic oxidation is a widely used surface modification strategy, which imposes an electrical flow between a titanium specimen and an electrode in an electrolyte, leading to anodic oxidation of the titanium specimen. The anodic oxidation of titanium implants can be conducted at low voltage, below the dielectric breakdown limit (potentiostatic method), or at high voltage (spark anodization), usually above 100 V (galvanostatic method) [41]. The principal aim of the anodic technique is to remove the native thin oxide layer and associated contaminants and to grow a tailored surface oxide layer with various properties. Hence, by controlling several parameters (including voltage, current, electrolyte, the addition of ions, PH, and temperature), oxidized titanium surfaces are obtained, with different micron and submicron porosities, varied surface oxide thicknesses, crystalline structure, and selected ion contents [41,42]. A prime example of commercially available oxidized implants is the TiUnite (NobelBiocare), characterized by a thick oxide layer (1–10 μm) that is moderately rough (Sa 1–1.5 μm), porous (0.06–12 μm surface porosity), crystalline (anatase and rutile phases), and contains phosphorus ion (3–6 At%) [[42], [43], [44]]. Previous experimental studies demonstrated that implants with anodically oxidized surfaces attenuate the initial inflammatory response and accelerate and enhance early coupled bone formation and remodeling at the interface [12,13,44,45]. These surface-driven events resulted in a higher proportion of mature, well-remodeled bone in contact with the surface and a higher degree of biomechanical stability and integration than machined implants [44,45]. The above-mentioned experimental findings [12,13,44,45] corroborate clinical studies in which oxidized implants have been shown to promote successful, faster, and more predictable osseointegration, even under local or systemic bone-compromising conditions [[46], [47], [48], [49]]. Therefore, an implant with an oxidized surface and a unified geometrical design would provide a standardized system to pinpoint specific factors that may influence the secondary ISQ values, particularly after the early healing period.

Resonance frequency analysis has been unquestionably unique and instrumental in assessing the primary implant stability and during clinical follow-up after implantation. It has also played an essential role in the decision-making for the immediate versus delayed loading protocol [19]. However, there is no consensus on the extent to which the factors mentioned above differ in their effect on the secondary ISQ values. This information will be valuable for appropriate case selection, the surgical procedure, and the loading protocol, at least for a specific type of dental implant. This retrospective clinical study aimed to determine the relationship between secondary implant stability, as measured by ISQ, and different parameters related to an oxidized implant. We hypothesized that using a multivariate regression model and a unified implant type would allow us to determine the influential effects of different implant- and protocol-related parameters on the secondary ISQ values while controlling for potential confounding effects among these factors or other patient-related factors.

## 2 Materials and methods

The present retrospective study conforms with the Helsinki Declaration for patients participating in clinical studies. The study was conducted under ethical approvals from the Kuwait University Health Sciences Center Ethical Committee (VDR/EC/3676/2020) and the institutional review board (IRB) at Imam Abdulrahman bin Faisal University (IRB-2021-02-494). The study followed the STROBE guidelines for observational cohort studies.

Electronic charts of patients who received implants at Kuwait University Dental Center from 2016 to 2020 were reviewed. Patients (age between 21 and 90) and implants fulfilling the following inclusion criteria were selected for further evaluation: all implants were placed by the same primary investigator (1st author); all implants were anodically oxidized (Tiunite) and with tapered design (NobelReplace) and conical connection (NobelReplace Conical Connection; Nobel Biocare AG, Zurich, Switzerland); patients categorized as ASA1 and ASA2; non-smoker and smoker (>10 cigarettes/day) as well as normoglycemic (HbA1C value < 5.7 %) or diabetic (but HbA1C value < 8 %) patients were included; patients with no absolute contraindications for implant therapy and patients who received RFA tests at the time of superstructure connection. On the other hand, the following exclusion criteria were used: patients who received extensive bone grafting before implant placement; patients with significant medical conditions that influence the process of healing; patients who did not receive an RFA test at the time of superstructure connection; and patient who were exposed to radiation therapy in the head and neck region.

All RFA measurements of the secondary implant stability were performed by the PI (1st author) at the time of superstructure connection, using 3rd generation Osstell™ (Osstell IDx; Osstell, Gothenburg, Sweden). Seven implant- and protocol-related factors were investigated in this study: Implant length (8.0 mm; 10.0 mm; 11.5 mm; 13.0 mm), platform diameter (3.5 mm; 4.3 mm; 5.0 mm), loading protocol (immediate loading; delayed loading), surgical protocol (one stage; two stages), need for simultaneous grafting procedure (non-grafted; grafted), achievement of primary stability (absence of detectable mobility upon implant insertion) (achieved; not achieved) [50] and insertion torque (<35Ncm; >35Ncm) [51,52].

### 2.1 Data management and statistical analysis

Power and sample sizes were determined based on the standardized mean of ISQ values reported in a previous relevant study [53], using G∗Power software for sample size calculation (G Power, version 3.1.9.7). For a type I error of 5 %, an effect size of 0.15, and a power of 80 %, a sample size of at least 270 implants (for implant level) and 100 patients (for patient level) was required. The final sample size was increased to 305 implants and 135 patients to account for potential missing data. The differences in the ISQ values were determined using the Mann–Whitney *U* test or the Kruskal–Wallis test. A multivariate linear regression model was employed to assess the relationship between the implant-related factors and the ISQ values while adjusting for the recorded patient-related factors (age, gender, smoking, diabetes, healing time, and implantation site). Data was analyzed at the patient level and implant level. For patient-level analysis, one implant was blindly selected from each patient having more than one implant. The blinded selection was performed by masking the ISQ result column in the Excel data file. Hence, our statistical analysis of the patient-level data was not based on the implants’ mean for a patient with more than one implant. Statistics were performed using SPSS 26.0 (IBM Corp., Armonk, NY, USA). Graphical data (frequency charts and boxplots) were generated using GraphPad Prism software, Version 9.0.

## 3 Results

A total of 135 patients who had received 305 implants were included, with frequencies and percentages of distribution of implants per patient as follows: single implant (59 patients; 44 %), two implants (38 patients; 28 %), three implants (14 patients; 10 %), four implants (13 patients; 10 %), five implants (3 patients; 2 %), six implants (5 patients; 4 %), seven implants (1 patient; 1 %), 11 implants (1 patient; 1 %) and 13 implants (1 patient; 1 %).

### 3.1 Patient-level

Table 1 presents the demographic characteristics of the study population. The mean follow-up time from implantation to secondary ISQ registration was 20.29 ± 8.83 weeks (median = 18 weeks; range 7–55 weeks) (Table 1). The population consisted of 63 % females and 37 % males; 18 % were smokers, and 16 % were diabetic (diagnosed diabetes with HbA1C <8 %). About 44 % of the implants were placed in the maxilla, and 81 % were in the posterior location. Among all quadrants, the highest percentage of received implants was in the posterior mandible (50 %), followed by the posterior maxilla (32 %) (Table 1).

**Table 1:** Study demographics.

Continuous variables		Patient-level	Implant-level
		Mean (STDEV) [Range]	Mean (STDEV) [Range]
Age		47.10 (15.17) [[22], [23], [24], [25], [26], [27], [28], [29], [30], [31], [32], [33], [34], [35], [36], [37], [38], [39], [40], [41], [42], [43], [44], [45], [46], [47], [48], [49], [50], [51], [52], [53], [54], [55], [56], [57], [58], [59], [60], [61], [62], [63], [64], [65], [66], [67], [68], [69], [70], [71], [72], [73], [74], [75], [76], [77], [78], [79]]	47.10 (15.17) [[22], [23], [24], [25], [26], [27], [28], [29], [30], [31], [32], [33], [34], [35], [36], [37], [38], [39], [40], [41], [42], [43], [44], [45], [46], [47], [48], [49], [50], [51], [52], [53], [54], [55], [56], [57], [58], [59], [60], [61], [62], [63], [64], [65], [66], [67], [68], [69], [70], [71], [72], [73], [74], [75], [76], [77], [78], [79]]
Time		20.29 (8.83) [[7], [8], [9], [10], [11], [12], [13], [14], [15], [16], [17], [18], [19], [20], [21], [22], [23], [24], [25], [26], [27], [28], [29], [30], [31], [32], [33], [34], [35], [36], [37], [38], [39], [40], [41], [42], [43], [44], [45], [46], [47], [48], [49], [50], [51], [52], [53], [54], [55]]	20.32 (9.05) [[5], [6], [7], [8], [9], [10], [11], [12], [13], [14], [15], [16], [17], [18], [19], [20], [21], [22], [23], [24], [25], [26], [27], [28], [29], [30], [31], [32], [33], [34], [35], [36], [37], [38], [39], [40], [41], [42], [43], [44], [45], [46], [47], [48], [49], [50], [51], [52], [53], [54], [55], [56], [57], [58], [59], [60], [61], [62], [63]]
**Dichotomous/categorical variables**		**Patient-level**	**Implant-level**
		***N* (%)∗**	***n* (%)**^**#**^
Gender	Male	50 (37 %)	127 (42 %)
	Female	85 (63 %)	178 (58 %)
Smoking	No	111 (82 %)	228 (75 %)
	Yes	24 (18 %)	77 (25 %)
Diabetes	No (HbA1C<5.7 %)	114 (84 %)	242 (79 %)
	Yes (HbA1C<8 %)	21 (16 %)	63 (21 %)
Jaw	Maxilla	60 (44 %)	134 (44 %)
	Mandible	75 (56 %)	171 (56 %)
Location	Anterior	25 (19 %)	52 (17 %)
	Posterior	110 (81 %)	253 (83 %)
Quadrant	Anterior maxilla	17 (13 %)	34 (11 %)
	Posterior maxilla	43 (32 %)	100 (33 %)
	Anterior mandible	8 (6 %)	18 (6 %)
	Posterior mandible	67 (50 %)	153 (50 %)

∗*N* and percentages (%) refer to number of patients in each dichotomous/categorical variable.

∗*n* and percentages (%) refer to number of implants in each dichotomous/categorical variable.

Concerning the implant dimensions, the 10.0 mm long implants represented 51 %, whereas the lowest percent was the 13 mm long (4 %) (Table 2). Implants with a 4.3 mm diameter constituted 36 %, whereas 3.5 mm and 5.0 mm represented 30 % and 35 %, respectively (Table 2). Regarding the implantation procedure and loading protocol, 72 % were placed using a two-stage procedure, and 73 % had a delayed loading protocol. Simultaneous grafting was used in 59 % of all patients (Table 2). At the implantation time, 72 % of implants demonstrated an insertion torque >35 Ncm, and primary stability was achieved in 85 % of the patients (Table 2).

**Table 2:** Implant-related variables and the corresponding Implant Stability Quotient (ISQ) values presented at patient and implant levels. Statistical significance P-value is presented according to the Kruskal-Wallis test (for multiple-group comparisons) and Mann-Whitney *U* test (for two-group comparisons).

Variables		Patient-level			Implant-level		
		N (%) ∗	ISQ (SD)	P-value	n (%)^#^	ISQ (SD)	P-value
Platform diameter	3.5 mm	40 (30 %)	72.09 (6.46)	<0.0001	71 (23 %)	72.78 (7.72)	<0.0001
	4.3 mm	48 (36 %)	78.00 (6.75)		141 (46 %)	77.82 (6.88)	
	5.0 mm	47 (35 %)	81.01 (5.61)		93 (31 %)	79.46 (6.52)	
Implant length	8.0 mm	21 (16 %)	79.98 (6.95)	0.07	55 (18 %)	77.47 (8.15)	0.59
	10.0 mm	69 (51 %)	77.61 (7.02)		160 (52 %)	77.18 (7.44)	
	11.5 mm	39 (29 %)	76.03 (7.35)		82 (27 %)	77.12 (6.86)	
	13.0 mm	6 (4 %)	72.58 (7.17)		8 (3 %)	74.56 (7.09)	
Surgical protocol	One stage	38 (28 %)	79.54 (7.39)	0.006	87 (29 %)	78.71 (7.97)	0.002
	Two stages	97 (72 %)	76.42 (6.99)		218 (71 %)	76.53 (7.08)	
Loading protocol	Immediate	36 (27 %)	76.93 (5.43)	0.28	71 (23 %)	76.60 (6.08)	0.12
	Delayed	99 (73 %)	77.43 (7.79)		234 (77 %)	77.32 (7.76)	
Grafting	No	55 (41 %)	77.79 (7.82)	0.33	123 (40 %)	78.46 (7.31)	0.01
	Yes	80 (59 %)	76.96 (6.80)		182 (60 %)	76.27 (7.35)	
Insertion torque	<35 Ncm	38 (28 %)	74.59 (6.89)	0.001	94 (31 %)	76.03 (6.74)	0.007
	>35 Ncm	97 (72 %)	78.36 (7.10)		211 (69 %)	77.65 (7.64)	
Primary stability	Not Achieved	20 (15 %)	73.43 (5.70)	0.001	43 (14 %)	74.88 (6.11)	0.003
	Achieved	115 (85 %)	77.97 (7.26)		262 (86 %)	77.52 (7.54)	

∗N and percentages (%) refer to number of patients in each dichotomous/categorical variable.

∗*n* and percentages (%) refer to number of implants in each dichotomous/categorical variable.

**Fig. 1:**
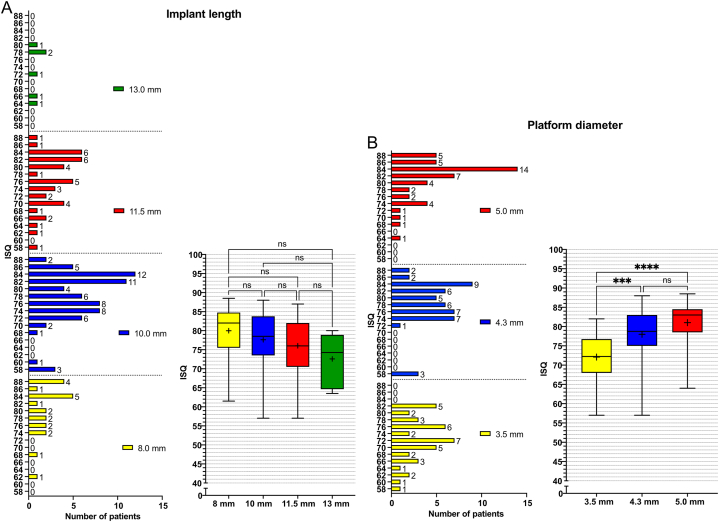
Implant platform diameter and implant length. The graphs show the frequency distribution and the corresponding boxplot of Implant Stability Quotient (ISQ) values regarding (A) the implant lengths (8, 10, 11.5, and 13 mm), and (B) platform diameter (3.5, 4.3, and 5.0 mm), presented at the patient level (N = 135). Statistical comparisons were performed using the Kruskal-Wallis test, followed by the Mann-Whitney *U* test. (∗∗∗∗) indicate the statistically significant differences (P < 0.0001); (∗∗∗) indicate the statistically significant differences (P < 0.001); ns: not significant.

**Fig. 2:**
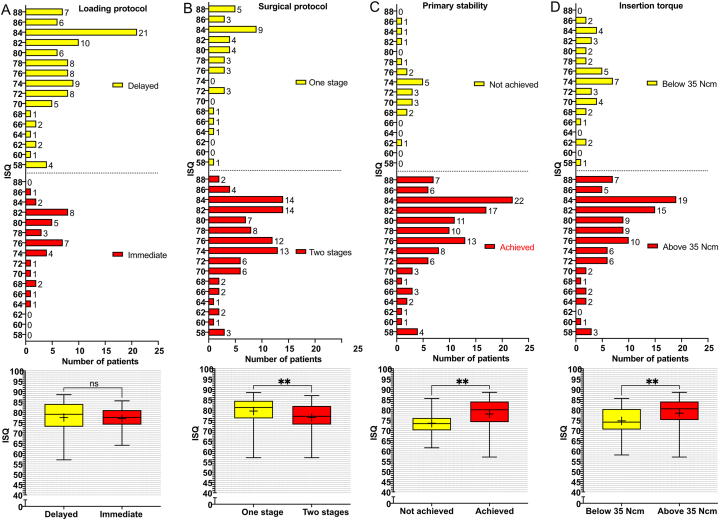
Protocols and primary stability. The graphs show the frequency distribution and the corresponding boxplot of Implant Stability Quotient (ISQ) values regarding (A) the loading protocol (delayed loading vs. immediate loading), (B) the surgical protocol (one stage vs. two stages), (C) the primary stability (achieved vs. not achieved), and (D) the insertion torque (below 35 Ncm vs. above 35 Ncm), presented at the patient level (N = 135). Statistical comparisons were performed using the Mann-Whitney *U* test. (∗∗) indicate the statistically significant differences (P < 0.01); ns: not significant.

**Fig. 3:**
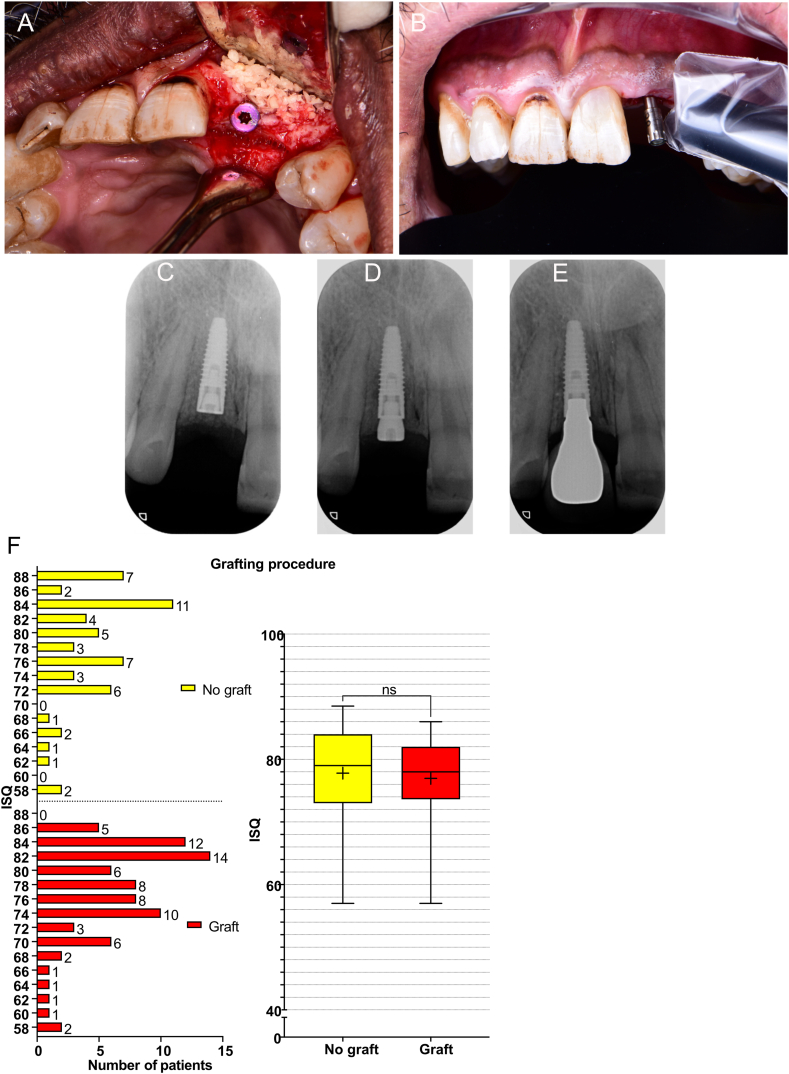
Grafting procedure. The upper panel shows selected images of implant placement with simultaneous grafting procedure (A) and resonance frequency analysis (RFA) of the same implant after the early healing phase at the time of superstructure connection. The apical radiographs show an example implant placed using a two-stage procedure and delayed loading at the time of placement (C), after the early healing phase (D), and after loading (E). The lower panel graphs show the frequency distribution and the corresponding boxplot of Implant Stability Quotient (ISQ) values regarding the grafting procedure (grafting vs. no grafting), presented at the patient level (N = 135). Statistical comparisons were performed using the Kruskal-Wallis test, followed by the Mann-Whitney *U* test. ns: not significant.

The effect of the implant-related factors on secondary ISQ values at the patient level was first evaluated individually using comparative analysis (Fig. 1, Fig. 2, Fig. 3). The mean endpoint ISQ for the 135 patients was 77.30 ± 7.22. There were no significant differences in the ISQ values neither between the different implant lengths (P = 0.07) (Fig. 1A) nor between the immediate and delayed loading protocols (P = 0.28) (Fig. 2A). The grafting versus no grafting procedures (Fig. 3A–E) did not reveal significant difference at the patient level (P = 0.33) (Fig. 3F). All other evaluated parameters revealed statistically significant differences. The platform diameter demonstrated the most robust significant difference (P < 0.0001) (Fig. 1B), where the diameter of 3.5 mm had significantly lower ISQ values as compared to 4.3 mm (P = 0.0002) and 5.0 mm (P < 0.0001) diameters. The difference between 4.3 mm and 5.0 mm diameters was insignificant (P = 0.06). Further, the one-stage protocol was associated with significantly higher ISQ as compared to the two-stage protocol (P = 0.006) (Fig. 2B). Implants that achieved primary stability (Fig. 2C) and implants that were inserted with >35 Ncm torque (Fig. 2D) showed significantly higher secondary ISQ compared with implants that did not achieve primary stability (P = 0.001) and implants inserted with <35 Ncm torque (P = 0.001), respectively.

A hierarchical multivariate linear regression model was built to determine the most significant predictor(s) for the secondary ISQ values (Table 3). The hierarchical multivariate regression was performed with two consecutive blocks of variables to adjust for the registered patient-related factors. The first block included the patient-related factors to be adjusted for (age, gender, diabetes, smoking, healing time, and implantation locations). The second block added the implant- and procedure-related factors (implant diameter, implant length, surgical protocol, loading protocol, simultaneous grafting, insertion torque, and primary stability) as the variables of interest and presented the final model after adjustment.

**Table 3:** Multivariate linear regression analysis. The analysis was conducted to determine the relationship between the Implant Stability Quotient (ISQ) as the dependent variable and the analyzed implant-related factors as the independent variables.

Block	Variables	Patient level				Implant level			
		B	SE	*P*	Adj R^2^	B	SE	*P*	Adj R^2^
1	age (years)	−0.06	0.04	0.17	0.12	−0.01	0.03	0.74	0.06
	Gender (Male; Female)	1.20	1.43	0.40		0.17	1.10	0.87	
	Diabetes (No; Yes)	0.58	0.74	0.19		0.39	1.13	0.73	
	Smoking (No; Yes)	−1.66	0.81	0.44		−1.59	0.77	0.11	
	Healing time (weeks)	0.10	0.07	0.13		0.09	0.05	0.06	
	Implant location (Mandible; Maxilla)	−2.94	1.21	0.01		−2.09	0.85	0.01	
	Implant location (Anterior; Posterior)	4.73	1.61	0.003		3.42	1.16	0.003	
2	age (years)	−0.04	0.04	0.32	0.25	−0.01	0.03	0.80	0.11
	Gender (Male; Female)	1.54	1.36	0.26		0.59	1.13	0.60	
	Diabetes (No; Yes)	0.73	1.66	0.66		0.59	1.11	0.80	
	Smoking (No; Yes)	−1.28	1.71	0.96		−1.18	1.27	0.35	
	Healing time (weeks)	0.10	0.06	0.11		0.08	0.05	0.09	
	Implant location (Mandible; Maxilla)	−0.39	1.24	0.75		−1.68	0.85	0.05	
	Implant location (Anterior; Posterior)	0.45	1.77	0.80		0.84	1.27	0.51	
	**Implant diameter (3.5; 4.3; 5.0)**	**5.25**	**1.17**	**<0.0001**		**4.28**	**0.87**	**<0.0001**	
	Implant length (8.0; 10.0; 11.5; 13.0)	−0.12	0.52	0.80		−0.42	0.33	0.73	
	Surgical protocol (1 stage; 2 stages)	−1.33	1.42	0.35		−0.83	1.07	0.44	
	Loading protocol (immediate; delayed)	0.75	1.41	0.59		0.07	1.06	0.50	
	Simultaneous grafting (no; yes)	0.26	1.33	0.85		−0.90	0.99	0.36	
	Insertion torque (<35Ncm; >35Ncm)	0.10	1.74	0.95		0.71	1.15	0.54	
	Primary stability (not achieved; achieved)	3.07	2.11	0.15		2.54	1.46	0.08	

Block 1 presents the patient-related factors (age, gender, diabetes, smoking, healing time, and implantation locations) that were adjusted for, whereas block 2 presents the final model after the adjustment. Significant relationship in the final model is highlighted in bold fonts.

Based on the regression analysis, the implant platform diameter was the only significant predictor for ISQ (B = 5.25 ± 1.03; P < 0.0001) after adjusting for the patient's related factors. The model's predictability was relatively high at the patient level, with an adjusted R square value of 0.25 (explaining 25 % of the variance in the ISQ values) (Table 3). The remaining regression analysis data, including the prior bivariate correlation and ANOVA, are provided in the Supplementary Information (Supplementary Tables S1, S3, and S4).

### 3.2 Implant-level

Table 1 presents the study demographic characteristics at the implant level, showing that 58 % and 42 % of implants were placed in female and male subjects, respectively. Non-smokers and normoglycemic patients received 75 % and 79 % of the total number of implants, respectively. Further, 56 % of all implants were placed in the mandible, and 83 % were placed in the posterior area. The frequency distribution of the implants was as follows: 50 % in the posterior mandible, 33 % in the posterior maxilla, 11 % in the anterior maxilla, and 6 % in the anterior mandible (Table 1).

Concerning the implant dimensions, the 10.0 mm long implants represented 52 %, whereas the lowest percentage was the 13 mm long (3 %) (Table 2). The frequencies of implant platform diameters were 23 %, 46 %, and 31 % for the 3.5-, 4.3- and 5.0-mm diameters, respectively (Table 2). Regarding the implantation procedure and loading protocol, 71 % were placed using a two-stage procedure, and 77 % had a delayed loading protocol. Simultaneous grafting was used in 60 % of all implants (Table 2). At the implantation time, 69 % of implants demonstrated an insertion torque >35 Ncm, and the primary stability was achieved in 86 % of the implants (Table 2).

The effect of the implant-related factors on secondary ISQ values at the implant level was first evaluated individually using comparative analysis (Supplementary Fig. S1-S3). The mean endpoint ISQ for the 305 implants was 77.15 ± 7.40. There were no significant differences in the ISQ values between the different implant lengths (P = 0.59) (Supplementary Fig. S1A) or between the immediate and delayed loading protocols (P = 0.12) (Supplementary Fig. S2A). All other evaluated parameters revealed statistically significant differences. The grafting versus no grafting procedures revealed a significant difference at the implant level with lower mean ISQ values in the grafted sites (P = 0.01) (Supplementary Fig. S1B). The platform diameter demonstrated the most robust significant difference (P < 0.0001) (Supplementary Fig. S1B), where the diameter of 3.5 mm revealed significantly lower ISQ values as compared to 4.3 mm (P < 0.0001) and 5.0 mm (P < 0.0001). The difference between 4.3 mm and 5.0 mm diameters was insignificant (P = 0.09). Further, the one-stage protocol was associated with significantly higher ISQ than the two-stage protocol (P = 0.002) (Supplementary Fig. S2B). Implants that achieved primary stability (Supplementary Fig. S2C) and implants that were inserted with >35 Ncm torque (Supplementary Fig. S2D) showed significantly higher secondary ISQ compared with implants that did not achieve primary stability (P = 0.003) and implants inserted with <35 Ncm torque (P = 0.007), respectively.

Using the regression analysis adjusted for the patient-related factors (as described above), the implant platform diameter was found to be the only significant predictor for ISQ (B = 4.28 ± 0.3; P < 0.0001) (Table 3). The model's predictability was lower at the implant level, with an adjusted R square value of 0.11 (explaining 11 % of the variance in the ISQ values) (Table 3). The remaining regression analysis data, including the prior bivariate correlation and ANOVA, are provided in the Supplementary Tables (Supplementary Tables S2, S3, and S4).

## 4 Discussion

The present study used comparative and correlational approaches to pinpoint which implant-related factor(s) significantly impact the secondary ISQ values (after healing) for a given implant type. We employed a regression model to account for potential confounding effects among the different factors and from patient-related factors. A major finding was that despite modest yet significant differences between several investigated parameters, these variations were omitted in the regression model, revealing that the platform diameter was the only factor that positively influenced the ISQ values for the early healing phase of osseointegration of the oxidized implant.

First, it is essential to highlight that the mean ISQ of this cohort was about 77 after a mean healing time of about five months. Moreover, the ISQ means remained high after the sub-groupings based on the different parameters, irrespective of being analyzed at the patient or implant levels. These findings support previous documentation of the present oxidized implant, experimentally and clinically, in achieving rapid osseointegration and firm stability in the recipient bone [44,45,54]. Further, the slight variations between the investigated variables do not seem to be of sizeable biological significance, at least within the study's time frame. This assumption is further supported by frequency distribution where the skewness of the data was generally towards the higher 10.13039/501100024890ISQ values beyond the means, even for the factors that showed relatively lower 10.13039/501100024890ISQ (i.e., implants with lower platform diameter, followed two-stage protocol, needed simultaneous graft, or had lower insertion torque or primary stability).

The present implant oxidized surface (TiUnite) is produced via spark anodization in an electrolyte containing Sulfuric and phosphoric acids (galvanostatic method), resulting in a thick TiO2 layer that is porous, moderately rough, crystalline and contains phosphorus ion [[42], [43], [44]]. The cellular and molecular response to this surface has been investigated in a series of in vivo studies. During the initial 24 h in rat tibia, the oxidized implant, compared to the machined implant, promoted a more significant influx of cells and higher expression of cell recruitment (CXCR4) and adhesion (integrins-β1, -β2 and -αv) markers [55]. During the period from 1 day to 28 days, the oxidized implant promoted higher expression of bone formation (Runx2, ALP, and OC) and bone remodeling/coupling (TRAP, CATK, RANK, RANKL, and OPG) genes and demonstrated a significant increase in biomechanical stability [44,45,56]. In contrast, the machined implant was associated with higher expression of proinflammatory cytokines (TNF-α and IL-1β) and showed non-significant increases in bone-implant contact and biomechanical stability [44,45,56].

It has also been shown that the secondary stability of similar oxidized implants can be further enhanced by incorporating magnesium ions in surface oxide [57]. Recently, it has been suggested that the potential mechanism for the reduced proinflammatory activity at oxidized implant may be related to an enhanced anti-inflammatory macrophage (M2) polarization driven by the oxidized surface [13]. In line with these findings, experimental implants oxidized at low voltages (potentiostatic method), having a thinner oxide layer but with a nanotubular pattern, promoted the bone formation and osteointegration in rodent models and enhanced the osteogenic differentiation of MSCs and osteoblastic cells in vitro [[58], [59], [60]]. Notably, the latter studies provided strong evidence of the role of the anodically oxidized surface with the nanotubular pattern in promoting anti-inflammatory M2 macrophage polarization. Moreover, it has been shown that incorporating ions (Zn, Mg, and Sr) further enhances the oxidized surface effects on osseointegration [[61], [62], [63]]. Finally, as previously observed with the oxidized surface produced at high voltage (with a think and microporous oxide layer), oxidized surfaces produced at low voltage (with thin nanotubular oxide layer) also demonstrated the association between the enhanced molecular response and the increased bone contact [64] and implant stability [65].

In the present study, the comparative analysis shows significant differences between the variables in all subgroups except for implant length and loading protocol subgroupings (and the grafting procedure at the patient level). These findings add to the controversial results reported in the literature where only comparative or bivariate correlations were conducted [[20], [21], [22], [23], [24], [25], [26], [27], [28], [29], [30], [31], [32], [33], [34], [35], [36], [37], [38], [39], [40]]. On the other hand, applying the multivariate regression model provided several advantages over the simple bivariate comparisons. Firstly, it considered the potential collinearity of the implant-/protocol-related factors, thus excluding factors with the highest variance inflation effect. Further, it allowed the adjustment of the model for the registered patient-related factors that have shown correlations with ISQ values in a preliminary univariate evaluation. For instance, the solely demonstrated predictor, the platform diameter, remained statistically significant after adjusting for the implant location. This finding suggests that the relationship between implant platform diameter and ISQ values is not confounded by the effect of having a higher number of wider implants in denser bone locations such as the anterior mandible. In addition, the regression model would allow for ranking the degree of prediction of each significant predictor in the model. Collectively, the overall significance of the model, based on the ANOVA, was found to be high. However, it only explained 24 % (at the patient level) and 11 % (at the implant level) of the changes in the ISQ values based on adjusted R^2^. Moreover, as only the platform diameter factor was found to significantly predict the secondary ISQ, the unstandardized B coefficient indicated that every unit change in the implant platform diameter can predict up to 5.7 units increase in ISQ values.

In contrast to comparative and correlation studies, fewer studies have employed regression models to determine the most influential factors for secondary ISQ measurement. One retrospective used a multivariate linear regression model to separately evaluate data obtained by three surgeons using either one or two implant types: one is blasted with zirconia beads and acid-etched (SICace implants), and the other is blasted with alumina particle and acid-etched (TSIII implants) [66]. The two implant types had a relatively varied surface roughness compared to the present oxidized implant, which has an average roughness of around 1.4 μm. According to their manufacturers’ information, the SICace implant has a roughness of 1.0 μm, whereas the TSIII implant conveys a higher roughness of 2.5–3.0 μm. In an almost identical scenario, only the implant platform diameter (3.5, 3.7, 4.0, 4.2, 4.5, 5.0 mm) demonstrated a positive significant prediction of the ISQ values during final restoration [66]. Otherwise, implant length, surgical protocol, and insertion torque did not reveal significant relationships with the secondary ISQ values [66]. The same research group in another study focusing only on the SICace implant demonstrated a similar finding of a potent effect of the platform diameter on ISQ values after healing. However, in the latter study, the insertion torque was also a significant positive predictor for RFA after healing [67]. Nonetheless, the unstandardized B coefficient for the insertion torque contribution was very low (B = 0.05), suggesting a modest prediction of the ISQ values during the final restoration. Notably, the unstandardized B coefficient for the implant platform diameter was found to be very high and comparable between the present (B = 4.45) and the previous (B = 3.5–4.19) [66,67] studies.

Interestingly, the length and the diameter exhibited variable contributions to ISQ measurement. Whereas no significant contribution of the implant length was observed, the width was found to be the most significant contributor, with the higher the diameter, the higher the ISQ. Despite controversial results that have been reported regarding the nominal effect of implant length and width on ISQ values, the present study is in complete agreement with previous studies that had specifically correlated the diameter (3.3, 4.1, and 4.8 mm) and length (6.0, 8.0, 10.0, 12.0 and 14.0 mm) of sandblasted, SLA Straumann implants, with the secondary ISQ (Osstell) as well as the damping capacity assessment (Periotest) over 1–6 years [68]. In the latter study, all correlational, comparative, and linear regression analyses demonstrated that the secondary ISQ measurement was only affected by the diameter, with higher ISQ values recorded for the wider implant, irrespective of the length [68]. In this context, the comparison analyses in the present and the previous [68] studies revealed that the significance was recorded for the lowest platform diameters (3.5 mm and 3.3 mm, respectively) versus the higher diameters in either study. In contrast, no significant difference could be found between the two higher diameters (i.e., 4.3 and 5.0 or 4.1 and 4.8, respectively).

In contrast, Shiffler and colleagues compared the ISQ values of oxidized implants with different lengths and diameters [69]. They concluded that no statistical difference exists between the evaluated lengths or diameters at placement and follow-up [69]. Notably, the implants used in their study were from the same manufacturer and had the same design and surface treatment as those used in our study. Nevertheless, it is noteworthy that in Shiffler et al.’s study, only two diameters were used (4.3 and 5.0) as opposed to our study, which included three diameters (3.5, 4.3, and 5.0). Interestingly, our study revealed the main difference between the implant with a 3.5 mm diameter and the other two with larger diameters, adding to the literature and Shiffler et al.’s study. Finally, Shiffler and colleagues only included two implant lengths, while our study included four, complimenting and adding to their findings. Regarding the implant diameter, the present clinical results, using oxidized implants, are closely similar to recent work in a dog model demonstrating lower ISQ values for sandblasted/acid-etched (SLA) implants with 3.3 mm diameter versus 4.1- and 4.8-mm implants, whereas no difference was found between 4.1- and 4.8-mm implants [35]. Notably, the latter experimental study revealed that the lower ISQ with the lowest diameter (3.3 mm) implant was persistent from baseline and throughout all study periods (4, 8, and 12 weeks).

Hitherto, the exact reason for the discrepancy between the implant length and platform diameter in their effect on ISQ remains to be determined. In a recent in vitro study, Gottlow and Sennerby concluded that the length has a stronger ISQ predictability than the diameter for blasted implants in polyurethane blocks with uniform density [70]. However, in addition to the in vitro nature of the study, the authors warranted further investigation using laminated blocks with graded density mimicking the natural bone [70]. Hence, it can be assumed that the increase in implant length or diameter would be associated with an increase in implant surface area in juxtaposition with the recipient bone. However, a plausible added effect for the wider implant diameter is that it may provide more contact with the denser bone in the crestal cortical region.

An outstanding question is what RFA represents concerning the process of osseointegration. From a clinical perspective, RFA remains the most convenient, non-invasive method to judge implant stability at any specific time point after implantation. On the other hand, from a fundamental scientific perspective, RFA has been questioned to be a true representative of the degree of bone-to-implant contact (BIC), which is the hallmark of osseointegration. For instance, experimental correlative studies [71,72] and studies that employed a regression model [73] refuted any significant relationship between the ISQ values and BIC%. In contrast, BIC% was found to be significantly predicted by the removal torque values of that implant [73]. In line with these histological findings, finite elemental analysis, which modeled a systematic increase in the contact between the implant and bone, showed that above a BIC value of 20 %, the ISQ values do not change significantly, suggesting a lack of sensitivity of RFA to further osseointegration beyond 20 % BIC [74]. These experimental findings support recent clinical evidence demonstrating a significant correlation between cortical bone contact to the implant and insertion torque [75]. On the contrary, such a correlation was not found with the secondary ISQ [75].

Although ISQ values do not correlate with BIC, they may reflect the degree of stiffness of the bone-implant unit [18,19,76]. Such a theory indicates that rigid continuity between the implant surface and the recipient bone may be responsible for making the bone and implant respond as one unit to the piezoelectric vibration. Although speculative, this assumption is supported by previous ultrastructural analysis of the bone interface with the oxidized implant, revealing, at a high-resolution level, a direct continuity between the implant surface and mineralized bone, filling the micron and sub-micron irregularities of the surface oxide layer [44,45]. Those findings may also explain the observed high ISQ values associated with the oxidized implants, which may have omitted any contributions of other factors apart from the implant platform diameter.

The present study investigated the effect of implant- and protocol-related factors on RFA using the same implant type so that the impact of variations of different implant designs and micron and submicron surface features would be eliminated. Moreover, the same surgeon, an expert in implant dentistry, has conducted all implant procedures, evaluations, and ISQ measurements, thus ensuring better consistency in implant placement and data collection. Nonetheless, a limitation of both aspects can, at least partly, attenuate the generalization of the study findings. Another limitation is the heterogeneity of some variables, such as the discrepancies in the number of patients treated under delayed loading versus immediate loading and the presence of cases treated with simultaneous grafting. In addition, the inclusion of smokers (18 %) and diabetic patients (16 %) poses a third limitation, given the known negative impact of those conditions on osteointegration. While the inclusion of smokers and diabetic patients was mainly to achieve the targeted sample size, studies on the early healing before final restoration (90 days after implantation) revealed that ISQ values were significantly higher in smokers versus non-smokers, especially for the implants with oxidized/TiUnite surface [77]. Further, considering the theory that RFA provides a direct measurement for bone stiffness [19], detailed ultrastructural and compositional characterizations of healing alveolar sockets revealed that the bone microstructure and composition of moderate-heavy smokers are comparable to those of never-smokers [78]. In the same context, the ISQ values for the secondary implant stability (12–16 weeks after implantation) were similarly high for sandblasted/acid-etched (SLA) and sandblasted/acid-etched/saline-stored (SLActive) implants, even in patients with poor glycemic control (type II diabetes) [79].

This study shows that wider platforms for the present implant system increase the secondary implant stability prior to final restoration. This effect would help improve implant stability in cases with reduced bone quality (e.g., soft bone). Moreover, predicting the degree of increase in ISQ values can be valuable when a specific ISQ range is sought (65 and above for immediate implant loading). Overall, the present findings would be valuable for appropriate case/implant selection, at least considering the present implant system. Further, as another clinical study using blasted/acid-etched implants demonstrated a similar finding that the implant width is the main predictor for the secondary ISQ, it is of interest to determine if other implant systems or implants with different surfaces would have similar or different predictors and predicting values for the secondary implant stability. Hence, prospective clinical studies are warranted to compare the predicting factors of ISQ for different implant systems using carefully selected inclusion criteria and advanced statistical models.

Secondary ISQ values of implants with oxidized/TiUnite surfaces appear to be higher when they have a wider platform diameter when placed using a single-stage approach, without grafting, or with insertion torque higher than 35 Ncm. However, considering the potential confounding effects and based on a multivariate regression model, it is concluded that among the different implant-related parameters, the platform diameter of the oxidized implant is the only significant positive predictor of the secondary ISQ values at the time of final restoration.

## CRediT authorship contribution statement

**Fawaz Alzoubi:** Writing – review & editing, Writing – original draft, Validation, Methodology, Investigation, Conceptualization. **Abdulkareem Abdullah Alhumaidan:** Writing – review & editing, Writing – original draft, Validation, Funding acquisition. **Hamad Saleh AlRumaih:** Writing – review & editing, Writing – original draft, Validation, Investigation. **Firas Khalid Alqarawi:** Writing – review & editing, Writing – original draft, Validation, Investigation. **Omar Omar:** Writing – review & editing, Writing – original draft, Visualization, Investigation, Formal analysis.

## 5 Ethics statement

The study was conducted under ethical approvals from the Kuwait University Health Sciences Center Ethical Committee (VDR/EC/3676/2020) and the institutional review board (IRB) at Imam Abdulrahman bin Faisal University (IRB-2021-02-494). The obtained IRB approval did not require informed patient consent for this retrospective study.

## Funding

The authors extend their appreciation to the Deputyship for Research & Innovation, Ministry of Education in Saudi Arabia, for funding this research work through the project number IF-2020-005-Dent at Imam Abdulrahman bin Faisal University/10.13039/100006582College of Dentistry. The funder had no role in this study's design, data collection, analysis, or reporting.

## Data availability statement

The data associated with this study has not been deposited into a publicly available repository. All data generated or analyzed during this study are included in this article and its supplementary material files. Further inquiries can be directed to the corresponding authors.

## Declaration of competing interest

The authors declare that they have no known competing financial interests or personal relationships that could have appeared to influence the work reported in this paper.
