# Supplementary material for: The relationship between the secondary implant stability quotient and oxidized implant-related factors: A retrospective study
Source: Heliyon. 2024 Oct 10;10(20):e39156. doi: 10.1016/j.heliyon.2024.e39156 (PMC11620092; doi:10.1016/j.heliyon.2024.e39156)
Supplement: Multimedia component 1 [file mmc1.docx]

**Supplementary Material**

**The relationship between the secondary implant stability quotient and oxidized implant-related factors: a retrospective study**

Fawaz Alzoubi, BDM, MA, EdD, Associate Professor^1*^, Abdulkareem Abdullah Alhumaidan, BDS, MA, PhD, Assistant Professor^2^, Hamad Saleh AlRumaih, BDS, MSD, PhD, Assistant Professor^3^, Firas Khalid Alqarawi, BDS, CAGS, PhD, Assistant Professor^3^, Omar Omar, BDS, PhD, Associate Professor ^3,*^

^1^Department of General Dental Practice, Faculty of Dentistry, Kuwait University, Kuwait

^2^Department of Preventive Dental Sciences, College of Dentistry, Imam Abdulrahman bin Faisal University, Dammam, Saudi Arabia

^3^Department of Substitutive Dental Sciences, College of Dentistry, Imam Abdulrahman bin Faisal University, Dammam, Saudi Arabia

^4^Department of Biomedical Dental Sciences, College of Dentistry, Imam Abdulrahman bin Faisal University, Dammam, Saudi Arabia

**Supplementary Figures (Implant level data)**


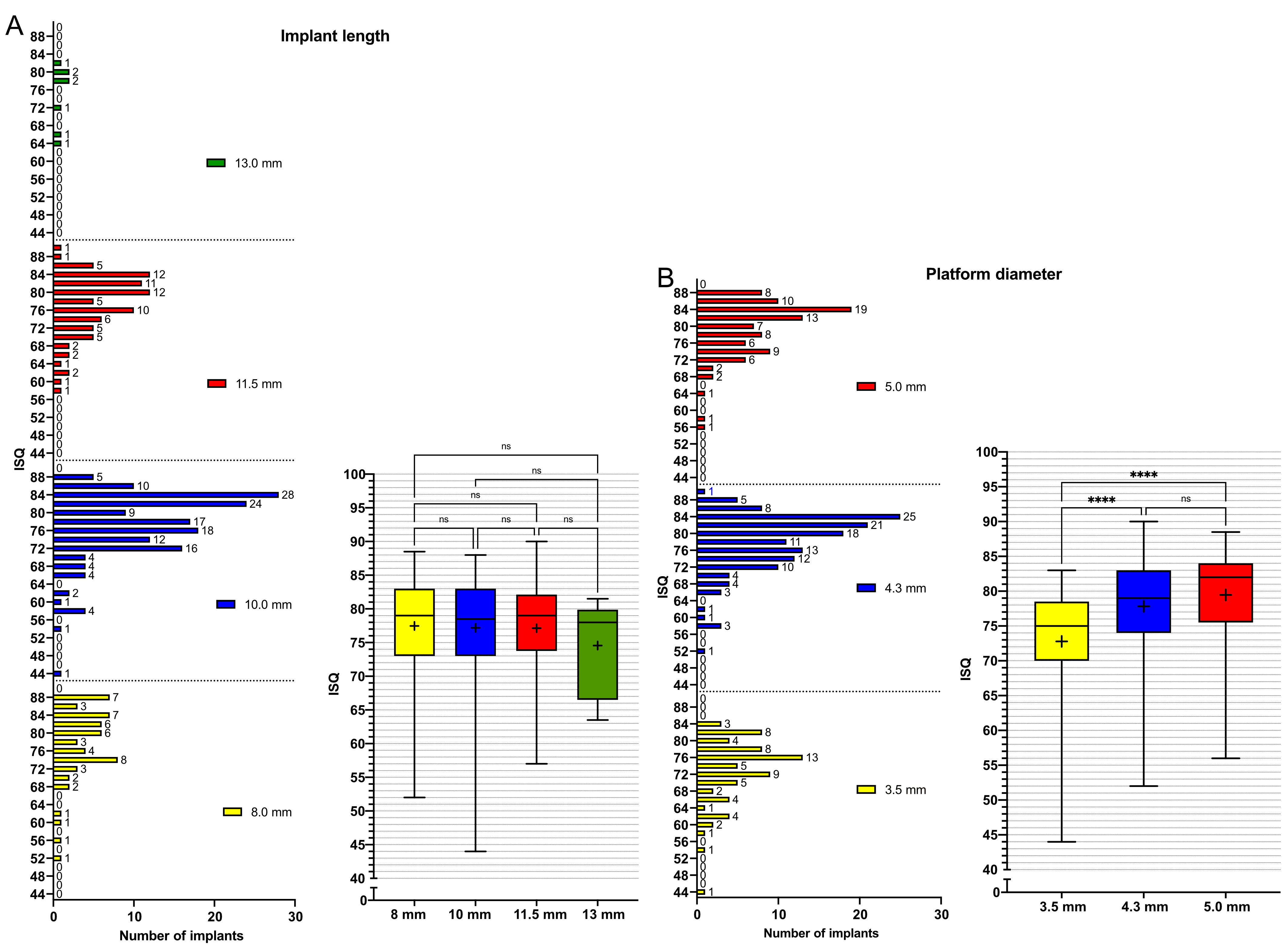


**Fig. S1.** Implant platform diameter and implant length. The graphs show the frequency distribution and the corresponding boxplot of Implant Stability Quotient (ISQ) values regarding (A) the implant lengths (8, 10, 11.5, and 13 mm), and (B) platform diameter (3.5, 4.3, and 5.0 mm), presented at the implant level (*n=305*). Statistical comparisons were performed using the Kruskal-Wallis test followed by Mann Whitney U test. (****) indicate the statistically significant differences (*P<0.0001*); ns: not significant.

**
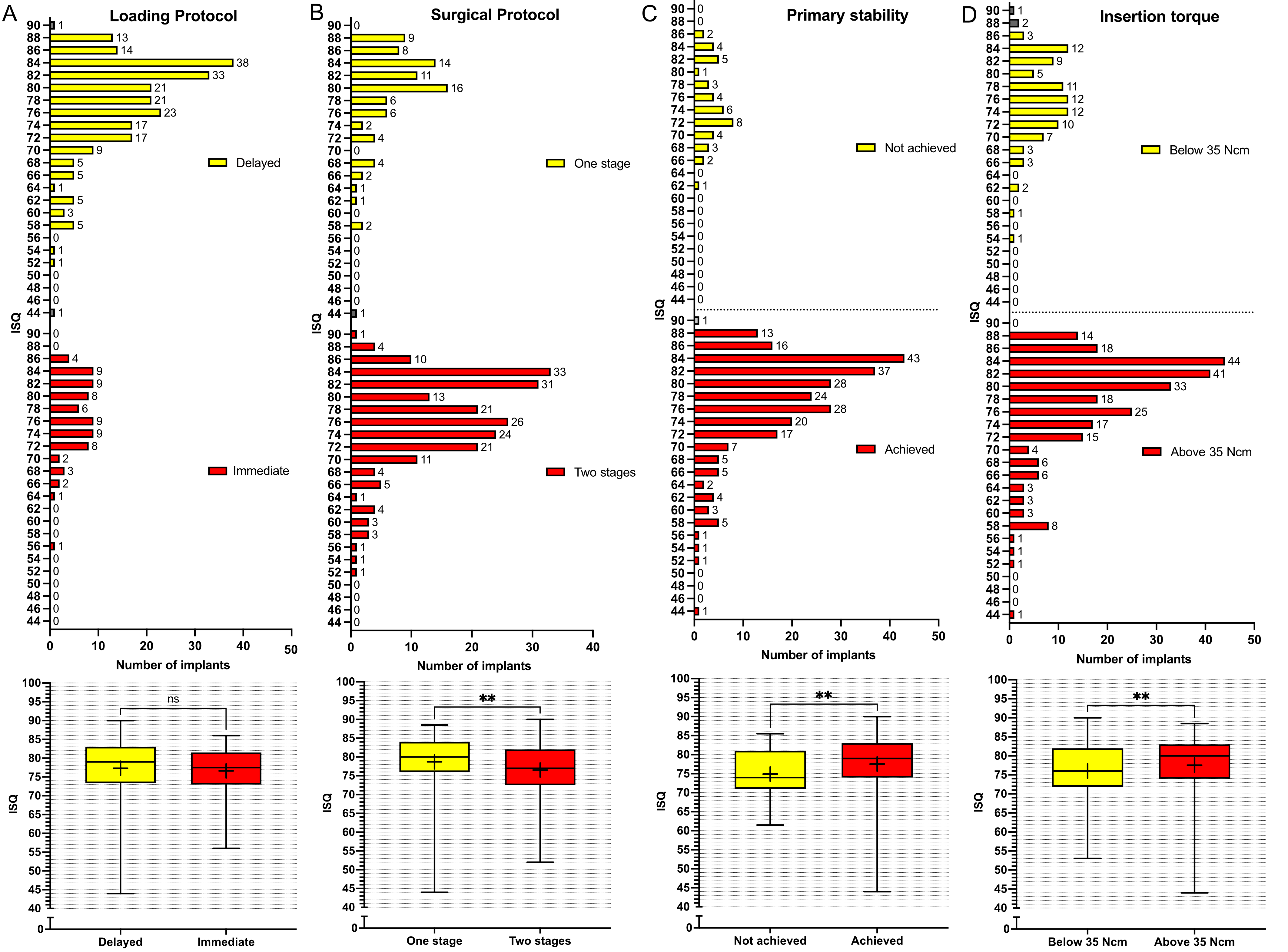
**

**Fig. S2.** Protocols and primary stability. The graphs show the frequency distribution and the corresponding boxplot of Implant Stability Quotient (ISQ) values regarding (A) the loading protocol (delayed loading vs. immediate loading), (B) the surgical protocol (one stage vs. two stages), (C) the primary stability (achieved vs. not achieved), and (D) the insertion torque (below 35 Ncm vs. above 35 Ncm), presented at the implant level (*n=305*). Statistical comparisons were performed using the Mann-Whitney U test. (**) indicate the statistically significant differences (*P<0.01*); ns: not significant.

**
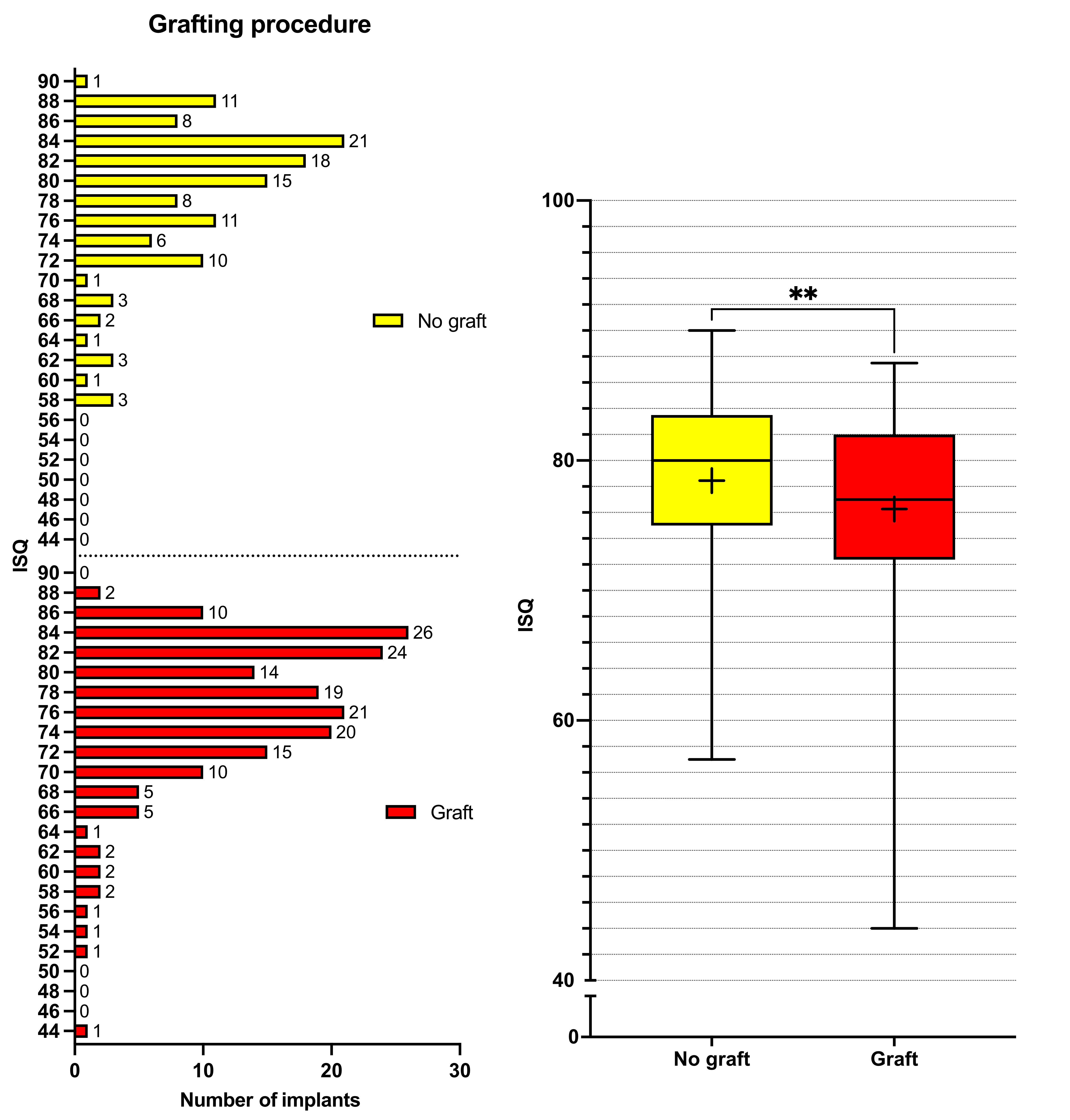
**

**Fig. S3.** Grafting procedure. The graphs show the frequency distribution and the corresponding boxplot of Implant Stability Quotient (ISQ) values regarding the grafting procedure (grafting vs. no grafting), presented at the implant level (*n=305*). Statistical comparisons were performed using the Kruskal-Wallis test followed by Mann Whitney U test. Statistical comparisons were performed using the Mann-Whitney U test. (**) indicate the statistically significant differences (*P<0.01*).

**Table S1:** Bivariate correlations at patient-level. The Pearson correlation coefficient *(r)* and significance level *(p)* are provided. The Table shows the bivariate correlation for the ISQ values with the different factors as well as in-between the different factors. Significant correlations are highlighted in bold fonts.

| Variables | | Age | Gender | Diabetes | Smoking | Healing time | Implant location1 | Implant location2 | Implant diameter | Implant length | Loading protocol | primary stability | Insertion torque | Surgical protocol | Grafting |
| --- | --- | --- | --- | --- | --- | --- | --- | --- | --- | --- | --- | --- | --- | --- | --- |
| ISQ | *(r)* | -0.1 | 0.13 | 0.03 | -0.07 | **0.18** | **-0.21** | **0.32** | **0.49** | **-0.22** | 0.03 | **0.22** | **0.23** | **-0.19** | -0.05 |
|  | *(p)* | 0.06 | 0.05 | 0.36 | 0.18 | **0.01** | **0.007** | **<0.0001** | **<0.0001** | **0.005** | 0.36 | **0.004** | **0.003** | **0.01** | 0.25 |
| Age | *(r)* |  | 0.02 | **0.37** | -0.12 | -0.10 | 0.05 | **-0.23** | -0.13 | 0.10 | **0.15** | -0.06 | -0.04 | 0.05 | 0.04 |
|  | *(p)* |  | 0.38 | **<0.0001** | 0.08 | 0.12 | 0.26 | **0.004** | 0.06 | 0.10 | **0.03** | 0.23 | 0.30 | 0.27 | 0.29 |
| Gender | *(r)* |  |  | 0.03 | **-0.52** | 0.0 | -0.117 | 0.06 | 0.01 | -0.01 | -0.02 | 0.11 | -0.003 | -0.003 | **0.20** |
|  | *(p)* |  |  | 0.35 | **<0.0001** | 0.29 | 0.08 | 0.21 | 0.43 | 0.43 | 0.39 | 0.09 | 0.48 | 0.48 | **0.008** |
| Diabetes | *(r)* |  |  |  | -0.09 | -0.03 | -0.01 | **-0.16** | 0.02 | -0.04 | 0.06 | 0.12 | 0.13 | 0.04 | 0.06 |
|  | *(p)* |  |  |  | 0.14 | 0.35 | 0.43 | **0.02** | 0.38 | 0.29 | 0.22 | 0.08 | 0.06 | 0.31 | 0.22 |
| Smoking | *(r)* |  |  |  |  | -0.04 | 0.05 | 0.02 | -0.06 | 0.03 | 0.11 | -0.02 | 0.07 | -0.01 | -0.12 |
|  | *(p)* |  |  |  |  | 0.30 | 0.27 | 0.39 | 0.24 | 0.33 | 0.09 | 0.39 | 0.19 | 0.45 | 0.07 |
| Healing time | *(r)* |  |  |  |  |  | 0.008 | **0.18** | 0.06 | -0.06 | -0.10 | 0.03 | 0.01 | **-0.18** | -0.005 |
|  | *(p)* |  |  |  |  |  | 0.46 | **0.01** | 0.22 | 0.24 | 0.12 | 0.35 | 0.44 | **0.01** | 0.47 |
| Implant location1 | *(r)* |  |  |  |  |  |  | **-0.22** | **-0.30** | **0.38** | 0.10 | **-0.21** | **-0.23** | -0.004 | 0.04 |
|  | *(p)* |  |  |  |  |  |  | **0.004** | **<0.0001** | **<0.0001** | 0.12 | **0.006** | **0.003** | 0.48 | 0.30 |
| Implant location2 | *(r)* |  |  |  |  |  |  |  | **0.53** | **-0.30** | -0.05 | 0.01 | **0.16** | **-0.17** | **-0.16** |
|  | *(p)* |  |  |  |  |  |  |  | **<0.0001** | **<0.0001** | 0.25 | 0.42 | **0.02** | **0.02** | **0.03** |
| Implant diameter | *(r)* |  |  |  |  |  |  |  |  | **-0.41** | -0.08 | 0.05 | **0.20** | -0.13 | **-0.17** |
|  | *(p)* |  |  |  |  |  |  |  |  | **<0.0001** | 0.16 | 0.25 | **0.008** | 0.06 | **0.02** |
| Implant length | *(r)* |  |  |  |  |  |  |  |  |  | **0.24** | -0.04 | -0.09 | -0.05 | 0.01 |
|  | *(p)* |  |  |  |  |  |  |  |  |  | **0.002** | 0.29 | 0.13 | 0.26 | 0.44 |
| Loading protocol | *(r)* |  |  |  |  |  |  |  |  |  |  | -0.03 | 0.00 | 0.11 | **0.36** |
|  | *(p)* |  |  |  |  |  |  |  |  |  |  | 0.35 | 0.47 | 0.08 | **<0.0001** |
| primary stability | *(r)* |  |  |  |  |  |  |  |  |  |  |  | **0.66** | **-0.26** | -0.006 |
|  | *(p)* |  |  |  |  |  |  |  |  |  |  |  | **<0.0001** | **0.001** | 0.47 |
| Insertion torque | *(r)* |  |  |  |  |  |  |  |  |  |  |  |  | **-0.35** | -0.08 |
|  | *(p)* |  |  |  |  |  |  |  |  |  |  |  |  | **<0.0001** | 0.16 |
| Surgical protocol | *(r)* |  |  |  |  |  |  |  |  |  |  |  |  |  | **0.35** |
|  | *(p)* |  |  |  |  |  |  |  |  |  |  |  |  |  | **<0.0001** |

Age (Years), Gender (Male; Female), Diabetes (No; Yes), Smoking (No; Yes) Healing time (Weeks), Implant location1 (Mandible; Maxilla), Implant location2 (Anterior; Posterior), Implant diameter (3.5; 4.3; 5.0), Implant length (8; 10; 11.5; 13), (Loading protocol (Immediate; Delayed), Primary stability (Not achieved; Achieved), Insertion Torque (<35Ncm; >35Ncm), Surgical protocol (1 stage; 2 stages), Grafting (No; Yes).

**Table S2:** Bivariate correlations at implant-level. The Pearson correlation coefficient *(r)* and significance level *(p)* are provided. The Table shows the bivariate correlation for the ISQ values with the different factors as well as in-between the different factors. Significant correlations are highlighted in bold fonts.

| Variables | | Age | Gender | Diabetes | Smoking | Healing time | Implant location1 | Implant location2 | Implant diameter | Implant length | Loading protocol | Primary stability | Insertion torque | Surgical protocol | Grafting |
| --- | --- | --- | --- | --- | --- | --- | --- | --- | --- | --- | --- | --- | --- | --- | --- |
| ISQ | *(r)* | -0.02 | 0.08 | 0.01 | **-0.10** | **0.13** | **-0.17** | **0.20** | **0.32** | -0.10 | 0.04 | **0.12** | **0.10** | **-0.13** | **-0.14** |
|  | *(p)* | 0.33 | 0.06 | 0.41 | **0.05** | **0.01** | **0.001** | **<0.0001** | **<0.0001** | 0.08 | 0.23 | **0.01** | **0.03** | **0.01** | **0.006** |
| Age | *(r)* |  | -0.02 | **0.38** | -0.06 | 0.02 | **0.11** | **-0.25** | -0.07 | 0.06 | **0.12** | -0.07 | 0.00 | **-0.11** | -0.003 |
|  | *(p)* |  | 0.32 | **<0.0001** | 0.14 | 0.35 | **0.02** | **<0.0001** | 0.09 | 0.12 | **0.01** | 0.09 | 0.49 | **0.02** | 0.48 |
| Gender | *(r)* |  |  | 0.004 | **-0.64** | 0.02 | -0.01 | 0.05 | 0.06 | **-0.15** | -0.007 | 0.07 | -0.01 | **0.14** | **0.20** |
|  | *(p)* |  |  | 0.47 | **<0.0001** | 0.34 | 0.39 | 0.15 | 0.13 | **0.003** | <0.45 | 0.08 | 0.38 | **0.006** | **<0.0001** |
| Diabetes | *(r)* |  |  |  | **-0.18** | 0.01 | 0.05 | **-0.15** | 0.05 | 0.009 | 0.02 | 0.04 | **0.11** | -0.07 | -0.02 |
|  | *(p)* |  |  |  | **0.001** | 0.40 | 0.17 | **0.003** | 0.15 | 0.43 | 0.32 | 0.22 | **0.02** | 0.10 | 0.32 |
| Smoking | *(r)* |  |  |  |  | -0.06 | 0.003 | 0.02 | **-0.11** | -0.02 | 0.01 | 0.04 | 0.06 | -0.08 | -0.09 |
|  | *(p)* |  |  |  |  | 0.14 | 0.48 | 0.34 | **0.02** | 0.36 | 0.36 | 0.24 | 0.14 | 0.07 | 0.05 |
| Healing time | *(r)* |  |  |  |  |  | 0.008 | **0.12** | 0.08 | 0.009 | -0.08 | -0.03 | -0.04 | **-0.17** | -0.03 |
|  | *(p)* |  |  |  |  |  | 0.44 | **0.01** | 0.06 | 0.43 | 0.08 | 0.29 | 0.21 | **0.001** | 0.28 |
| Implant location-1 | *(r)* |  |  |  |  |  |  | **-0.19** | **-0.17** | **0.18** | 0.02 | **-0.15** | **-0.16** | -0.04 | 0.001 |
|  | *(p)* |  |  |  |  |  |  | **<0.0001** | **0.001** | **0.01** | 0.31 | **0.004** | **0.002** | 0.24 | 0.49 |
| Implant location-2 | *(r)* |  |  |  |  |  |  |  | **0.45** | **-0.11** | **-0.10** | 0.04 | 0.05 | **-0.11** | **-0.17** |
|  | *(p)* |  |  |  |  |  |  |  | **<0.0001** | **0.02** | **0.03** | 0.23 | 0.16 | **0.02** | **0.001** |
| Implant diameter | *(r)* |  |  |  |  |  |  |  |  | 0.009 | -0.05 | 0.04 | **0.15** | **-0.15** | **-0.24** |
|  | *(p)* |  |  |  |  |  |  |  |  | 0.43 | 0.17 | 0.23 | **0.003** | **0.004** | **<0.0001** |
| Implant length | *(r)* |  |  |  |  |  |  |  |  |  | -0.06 | -0.04 | 0.002 | -0.003 | -0.08 |
|  | *(p)* |  |  |  |  |  |  |  |  |  | 0.13 | 0.20 | 0.48 | 0.48 | 0.06 |
| Loading protocol | *(r)* |  |  |  |  |  |  |  |  |  |  | -0.06 | 0.01 | **0.15** | **0.39** |
|  | *(p)* |  |  |  |  |  |  |  |  |  |  | 0.12 | 0.39 | **0.003** | **<0.0001** |
| primary stability | *(r)* |  |  |  |  |  |  |  |  |  |  |  | **0.58** | **-0.25** | 0.03 |
|  | *(p)* |  |  |  |  |  |  |  |  |  |  |  | **<0.0001** | **<0.0001** | 0.29 |
| Insertion torque | *(r)* |  |  |  |  |  |  |  |  |  |  |  |  | **-0.37** | -0.08 |
|  | *(p)* |  |  |  |  |  |  |  |  |  |  |  |  | **<0.0001** | 0.06 |
| Surgical protocol | *(r)* |  |  |  |  |  |  |  |  |  |  |  |  |  | **0.35** |
|  | *(p)* |  |  |  |  |  |  |  |  |  |  |  |  |  | **<0.0001** |

Age (Years), Gender (Male; Female), Diabetes (No; Yes), Smoking (No; Yes) Healing time (Weeks), Implant location1 (Mandible; Maxilla), Implant location2 (Anterior; Posterior), Implant diameter (3.5; 4.3; 5.0), Implant length (8; 10; 11.5; 13), (Loading protocol (Immediate; Delayed), Primary stability (Not achieved; Achieved), Insertion Torque (<35Ncm; >35Ncm), Surgical protocol (1 stage; 2 stages), Grafting (No; Yes).

**Table S3:** Regression model summary at patient and implant levels.

|  | **Block** | **R** | **R Square** | **Adjusted R Square** | **Std. Error of Estimate** | **Change Statistics** | | | | |
| --- | --- | --- | --- | --- | --- | --- | --- | --- | --- | --- |
|  |  |  |  |  |  | **R Square Change** | **F Change** | **df1** | **df2** | **Sig. F Change** |
| **Patient level** | 1 | 0.41 | 0.17 | 0.12 | 6.75 | 0.17 | 3.70 | 7 | 127 | 0.001 |
|  | 2 | 0.57 | 0.33 | 0.25 | 6.24 | 0.16 | 4.11 | 7 | 120 | <0.0001 |
| **Implant level** | 1 | 0.29 | 0.08 | 0.06 | 7.16 | 0.08 | 3.92 | 7 | 297 | 0.0001 |
|  | 2 | 0.39 | 0.15 | 0.11 | 6.94 | 0.07 | 3.64 | 7 | 290 | 0.001 |

Block 1 presents the regression model summary the patient-related factors (age, gender, diabetes, smoking, healing time, and implantation locations) that were adjusted for, whereas block 2 presents the summary for the final model after the adjustment. Significant relationship in the final model is highlighted in bold fonts.

**Table S4:** ANOVA in the regression analysis at patient and implant levels.

|  | **Block** | **Model** | **Sum of Squares** | **df** | **Mean Square** | **F** | **Sig.** |
| --- | --- | --- | --- | --- | --- | --- | --- |
| **Patient level** | 1 | Regression | 1183.39 | 7 | 169.05 | 3.705 | 0.001 |
|  |  | Residual | 5795.25 | 127 | 45.63 |  |  |
|  |  | Total | 6978.64 | 134 |  |  |  |
|  | 2 | Regression | 2304.91 | 14 | 164.63 | 4.22 | <0.001 |
|  |  | Residual | 4673.73 | 120 | 38.94 |  |  |
|  |  | Total | 6978.64 | 134 |  |  |  |
| **Implant level** | 1 | Regression | 1410.74 | 7 | 201.53 | 3.92 | <0.001 |
|  |  | Residual | 15233.92 | 297 | 51.29 |  |  |
|  |  | Total | 16644.66 | 304 |  |  |  |
|  | 2 | Regression | 2641.57 | 14 | 188.68 | 3.90 | <0.001 |
|  |  | Residual | 14003.08 | 290 | 48.28 |  |  |
|  |  | Total | 16644.66 | 304 |  |  |  |

Block 1 presents the ANOVA results in the regression model for the patient-related factors (age, gender, diabetes, smoking, healing time, and implantation locations) that were adjusted for, whereas block 2 presents the ANOVA results in the regression for the final model after the adjustment. Significant relationship in the final model is highlighted in bold fonts.
